# Epithelial–mesenchymal transition confers resistance to selective FGFR inhibitors in SNU-16 gastric cancer cells

**DOI:** 10.1007/s10120-014-0444-1

**Published:** 2014-11-19

**Authors:** Paulina Grygielewicz, Barbara Dymek, Anna Bujak, Pawel Gunerka, Aleksandra Stanczak, Monika Lamparska-Przybysz, Maciej Wieczorek, Karolina Dzwonek, Daria Zdzalik

**Affiliations:** 1 Innovative Drugs R&D Department, Celon Pharma Inc., Mokra 41a, 05-092 Lomianki/Kielpin, Poland; 2 Postgraduate School of Molecular Medicine, Zwirki i Wigury 61, 02-091 Warsaw, Poland; 3 Department of Medical Biotechnology, Medical University of Lodz, Al. Kosciuszki 4, 90-419 Łodz, Poland; 4 Department of Immunology, Center for Biostructure Research, Medical University of Warsaw, Banacha 1a, F Building, 02-097 Warsaw, Poland

**Keywords:** Fibroblast growth factor receptor, Epithelial–mesenchymal transition, Drug resistance, Fibroblast growth factor receptor inhibitor, AZD4547, BGJ398

## Abstract

**Background:**

Up to 10 % of primary gastric cancers are characterized by *FGFR2* amplification, and fibroblast growth factor receptor (FGFR) inhibitors may represent therapeutic agents for patients with these malignancies. However, long-term benefits of the treatment might be limited owing to the occurrence of drug resistance.

**Methods:**

To investigate the mechanisms of resistance to selective FGFR inhibitors, we established three *FGFR2*-amplified SNU-16 gastric cancer cell lines resistant to AZD4547, BGJ398, and PD173074, respectively.

**Results:**

The resistant cell lines (SNU-16R) demonstrated changes characteristic of epithelial-to-mesenchymal transition (EMT). In addition, they displayed loss of expression of FGFR2 and other tyrosine kinase receptors concurrent with activation of downstream signaling proteins and upregulation of the transforming growth factor β (TGF-β) level. However, treatment of parental SNU-16 cells with TGF-β_1_ did not evoke EMT, and pharmacological inhibition of TGF-β receptor I was not sufficient to reverse EMT changes in the resistant cells. Finally, we showed that the SNU-16R cell lines were sensitive to the human epidermal growth factor receptor 2 inhibitor mubritinib and the heat shock protein 90 inhibitor AUY922.

**Conclusion:**

In conclusion, we provide experimental evidence that EMT-mediated resistance might emerge in gastric cancer patients following treatment with FGFR inhibitors, and mubritinib or AUY922 treatment may be an alternative therapeutic strategy for these patients.

**Electronic supplementary material:**

The online version of this article (doi:10.1007/s10120-014-0444-1) contains supplementary material, which is available to authorized users.

## Introduction

The fibroblast growth factor (FGF) receptors (FGFRs) constitute one of the most extensively studied novel therapeutic targets in the field of anticancer drug development. The FGFR family comprises four members (FGFR1–FGFR4) which serve as high-affinity receptors for 22 different FGF ligands [1]. The FGF/FGFR pathway plays an important role in a myriad of key cellular processes, such as cell proliferation, differentiation, migration, survival, and angiogenesis [1]. Aberrant activation of FGFR kinases owing to gene amplification, chromosomal translocation, or gain-of-function mutation has been implicated in the pathogenesis of a variety of human cancers, including gastric cancer [2], breast cancer [3], bladder cancer [4], endometrial cancer [5], squamous cell lung cancer [6], multiple myeloma [7], and glioblastoma [8].

Since aberrantly activated FGFR kinases serve as an oncogenic “driver,” a great number of FGFR inhibitors are currently in clinical development. However, the vast majority of them are multikinase inhibitors such as dovitinib, nintedanib, or ponatinib [9]. To date, no selective small-molecule FGFR inhibitor has been approved for clinical use. The first selective FGFR inhibitor developed was PD173074, which, despite its high selectivity and cellular activity, has never entered clinical use [10]. At present, only a few selective FGFR inhibitors are under clinical investigation, and the two in the most advanced stage are AZD4547 and BGJ398, developed by AstraZeneca and Novartis, respectively [11, 12].

According to the World Health Organization, gastric cancer accounts for nearly one in ten of all deaths of cancer patients [13]. Mean survival for patients with stage IV metastatic gastric cancer is only 10 months [14]. Given this, there is an urgent need to improve gastric cancer therapy. *FGFR2* amplification occurs in 3–10 % of primary gastric cancers, and has been reported to be more frequent in the undifferentiated diffuse subtype [2]. The effectiveness of FGFR inhibitors against gastric cancer in vitro and in vivo suggests that the use of these compounds may be a therapeutic strategy for the selected group of patients with *FGFR2*-amplified gastric cancer [15, 16]. However, from experience with other kinase inhibitors used in clinical practice, there is a high probability that resistance will develop in patients following FGFR inhibitor treatment.

To date, there is a lack of clinical data concerning drug resistance in FGFR-inhibitor-based therapy. Nevertheless, recently published results from in vitro models cast some light on the possible mechanisms of resistance to FGFR inhibitors. It has been shown that the heterozygous gatekeeper mutation FGFR3 V555M emerged as a mechanism of acquired resistance to the selective FGFR inhibitor AZ12908010 in KMS-11 myeloma cells, and the resistant cells were cross-resistant to other FGFR inhibitors, AZD4547 and PD173074 [17]. Additionally, several activating mutations were identified in FGFR2-expressing Ba/F3 cells treated with high concentrations of dovitinib. Recognized mutations conferred cross-resistance to the selective FGFR inhibitor PD173074 but not to the multikinase inhibitor ponatinib, whose inhibitory activity was affected only by the V565I gatekeeper mutation [18]. In addition to secondary mutations in the kinase domain, resistance to kinase inhibitors may also occur through activation of alternative signaling pathways. With use of a high-throughput “secretome” screening platform, it has been established that ligand-mediated activation of compensatory kinases such as epidermal growth factor receptor (EGFR), human EGFR (HER), or MET may compensate for loss of FGFR kinase activity [19]. Additionally, using parallel RNA interference genetic screens, Herrera–Abreu et al. [20] discovered that EGFR is a key mediator of resistance in FGFR3-dependent cancer cells.

Given this, to predict the mechanisms of acquired resistance which may emerge in patients with FGFR2-dependent gastric cancer following treatment with selective FGFR inhibitors, we generated three SNU-16 cell lines resistant (SNU-16R) to AZD4547, BGJ398, and PD173074, respectively. It has been well established that SNU-16 cells harbor *FGFR2* amplification, and thereby they are highly sensitive to FGFR inhibitors in vitro and in vivo [2, 21–23]. However, long-term exposure to increasing concentrations of selected inhibitors induced resistance accompanied by loss of FGFR2 expression and epithelial-to-mesenchymal transition (EMT), a phenomenon that has previously been shown to be involved in resistance to other kinase inhibitors [24–27]. Finally, on the basis of our results, we propose possible therapeutic strategies to overcome EMT-mediated resistance to FGFR inhibitors.

## Materials and methods

### Compounds and cell lines

AT9283, AZD4547, AZD6244, BGJ398, crizotinib, pictilisib, ibrutinib, PD173074, ruxolitinib, TAE684, and gandotinib were all purchased from Selleck Chemicals, AUY922, dovitinib, everolimus, gefitinib, mubritinib, saracatinib, sunitinib, and ZSTK474 were from LC Laboratories, CH5424802 was from Active Biochem, SB525334 was from Tocris, and fedratinib was from Axon Medchem. The human gastric cancer cell line SNU-16 was obtained from ATCC and was cultured in RPMI 1640 medium supplemented with 10 % fetal bovine serum according to the manufacturer’s instructions.

### Generation of AZD4547-, BGJ398-, and PD173074-resistant SNU-16 cells

To generate drug-resistant cells, SNU-16 cells were exposed to increasing concentrations of AZD4547, BGJ398, or PD173074 for 1 month. The concentration of each inhibitor was doubled at every second passage to a final concentration of 0.6 µM for AZD4547 and 1.2 µM for BGJ398 and PD173074. The resistant cells were cultured until they again had growth kinetics similar to the growth kinetics of untreated parental cells.

### Treatment of parental SNU-16 cells with transforming growth factor β_1_

SNU-16 cells were cultured in a medium containing human recombinant transforming growth factor (TGF)-β_1_ (Merck Millipore) in a final concentration of 0.5 ng/ml. Fresh TGF-β_1_ was added every 72 h for 3 weeks.

### Cell viability assays

For viability tests, 6 × 10^3^ cells per well were seeded in 96-well plates and exposed to increasing doses of the tested compounds. After 72 h of drug treatment, cell viability was determined by the ATPlite assay (Perkin Elmer) according to the manufacturer’s instructions.

Half maximal inhibitory concentrations (IC_50_) of mubritinib were calculated with GraphPad Prism using the sigmoid dose–response function. All experiments were performed at least twice.

### Immunoblot analysis

SNU-16 parental and resistant cells were seeded in six-well plates at a density of 0.5 × 10^6^ cells per milliliter in an inhibitor-free medium. After 24 h, cells were lysed and the level of proteins was examined by Western blotting according to the protocols provided by the antibody suppliers. The antibodies against MET, EGFR, HER2, extracellular-signal-regulated kinase (ERK), phosphorylated AKT, phosphorylated EGFR, phosphorylated ERK (pERK), phosphorylated FGFR, phosphorylated FGFR substrate, and phosphorylated signal transducer and activator of transcription (STAT3) were purchased from Cell Signaling Technology, β-tubulin and AKT were from Millipore, E-cadherin, STAT3, and β-catenin were from BD Biosciences, FGFR2 was from Abnova, and vimentin was from Calbiochem. All experiments were performed at least twice.

### Phosphoprotein array analysis

SNU-16 parental and resistant cells were seeded in T25 bottles at a density of 1.5 × 10^6^ cells per bottle in an inhibitor-free medium. The assay was conducted in accordance with the commercial protocol of the PathScan^®^ receptor tyrosine kinase signaling antibody array kit (Cell Signaling Technology).

## Results

### SNU-16R cell lines exhibited a substantial decrease in sensitivity to all FGFR inhibitors tested

**Fig. 1:**
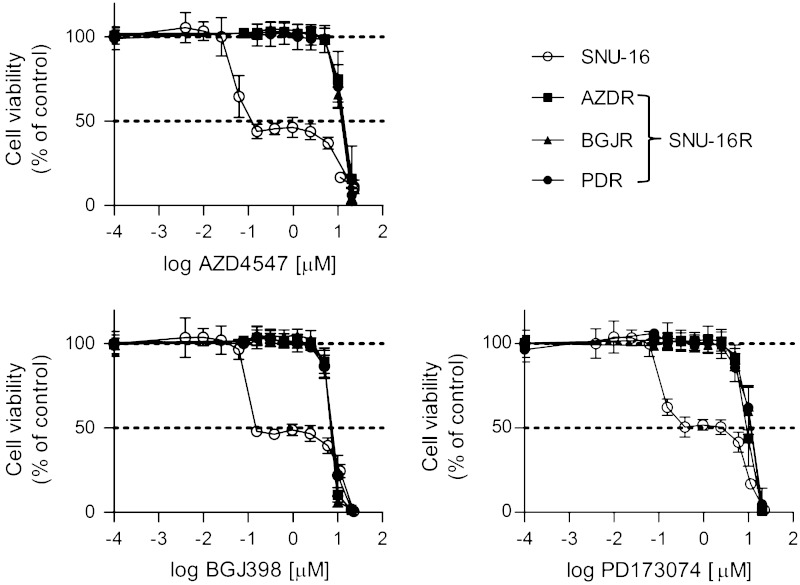
Sensitivity of parental SNU-16 and resistant SNU-16 (*SNU-16R*) cells to selective fibroblast growth factor receptor (FGFR) inhibitors. Cells were treated with increasing concentrations of AZD4547, BGJ398, or PD173074 for 72 h, and cell viability was measured with the ATPlite assay. Each data point represents the mean of two independent triplicate measurements, and *error bars* indicate the standard deviation. *AZDR* SNU-16 cell line resistant to AZD4547, *BGJR* SNU-16 cell line resistant to BGJ398, *PDR* SNU-16 cell line resistant to PD173074

To generate SNU-16 gastric cancer cell lines resistant to AZD4547 (AZDR), BGJ398 (BGJR), and PD173074 (PDR), SNU-16 cells were cultured with increasing concentrations of the respective FGFR inhibitor. In the viability assay, the parental cell line was shown to be sensitive to FGFR inhibitors, with 50 % inhibition of cell viability at a concentration of approximately 150 nM for AZD4547 and BGJ398, and 380 nM for PD173074 (Fig. 1). The observed growth inhibition was dose independent in range from 0.153 to 6 μM, and the viability of SNU-16 cells was completely inhibited only at high micromolar concentrations of the FGFR inhibitors. Given this, we were unable to fit sigmoid dose–response curves to the data and calculate true IC_50_ values. Consistent with the ability of resistant cells to grow at high concentrations of a particular FGFR inhibitor, AZDR, BGJR, and PDR cells were highly resistant to AZD4547, BGJ398, or PD173074, respectively, with an IC_50_ of approximately 10 μM (Fig. 1). Moreover, each resistant cell line exhibited cross-resistance to all FGFR inhibitors tested, and the resistance to AZD4547, BGJ398, and PD173074 was comparable among all resistant cell lines. Altogether, the substantial decrease in sensitivity of SNU-16R cell lines to FGFR inhibitors observed in the cell viability assay confirmed the emergence of drug resistance in SNU-16R cells.

### SNU-16R cells lose their dependency on FGFR2 signaling and display alterations in other signaling pathways

**Fig. 2:**
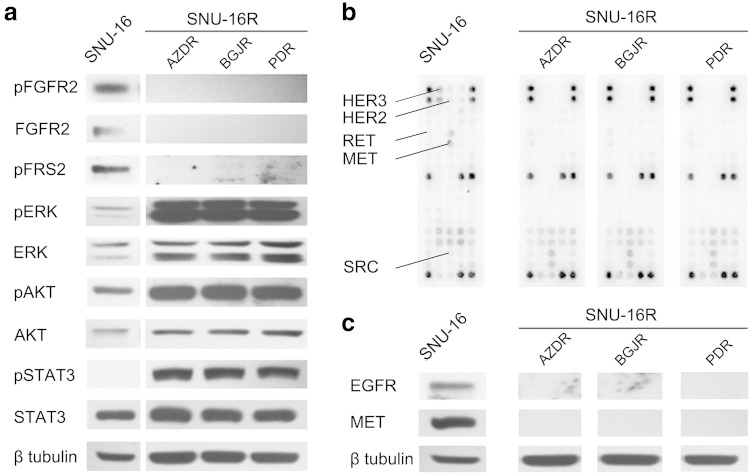
Alterations in signaling pathways in resistant SNU-16 (*SNU-16R*) cell lines. Parental SNU-16 and SNU-16R cells were incubated in inhibitor-free medium for 24 h, and then cell lysates from each cell line were subjected to immunoblot analysis. A representative result from one of two separate experiments is shown. **a** Immunoblot analysis of FGFR-dependent signaling and downstream proteins. **b** Analysis of phosphorylation status of key signaling molecules assessed by the PathScan^®^ receptor tyrosine kinase signaling antibody array. **c** Immunoblot analysis of the expression of the receptors epidermal growth factor receptor (*EGFR*) and MET. *AZDR* SNU-16 cell line resistant to AZD4547, *BGJR* SNU-16 cell line resistant to BGJ398, *ERK* extracellular-signal-regulated kinase, *FGFR* fibroblast growth factor receptor, *HER2* human epidermal growth factor receptor 2, *HER3* human epidermal growth factor receptor 3, *PDR* SNU-16 cell line resistant to PD173074, *pERK* phosphorylated extracellular-signal-regulated kinase, *pFGFR* phosphorylated fibroblast growth factor receptor, *pFRS2* phosphorylated fibroblast growth factor receptor substrate 2, *pSTAT3* phosphorylated signal transducer and activator of transcription 3, *STAT3* signal transducer and activator of transcription 3

To examine the mechanism of resistance to FGFR inhibitors in SNU-16R cell lines, firstly we determined the status of FGFR2 signaling by immunoblot analysis. It revealed that the level of FGFR2 was undetectable in resistant cells in contrast to parental ones (Fig. 2a). Consistent with these findings, the decrease of phosphorylation of phosphorylated FGFR substrate 2, a direct downstream target of FGFR2, was also observed (Fig. 2a). Given this, all SNU-16R cell lines became independent of FGFR2 signaling. However, despite loss of FGFR2 expression, phosphorylation of downstream proteins such as ERK, AKT, and STAT3 was upregulated in AZDR, BGJR, and PDR cells (Fig. 2a). This suggested that activation of alternative pathways could be responsible for the observed increase in phosphorylation status of downstream proteins.

To verify whether acquired resistance of AZDR, BGJR, and PDR cells is the effect of the induction of an alternative signaling pathway, we used the slide-based PathScan^®^ receptor tyrosine kinase signaling antibody array kit. Phosphoprotein array analysis confirmed that all SNU-16R cell lines exhibit alterations in diverse signaling pathways in comparison with parental SNU-16 cells. We found a slight increase in the phosphorylation status of SRC and RET concomitantly with downregulation of phosphorylated MET and phosphorylated HER3 in all three SNU-16R cell lines compared with wild-type SNU-16 cells (Fig. 2b). Additional immunoblot analysis showed that the decrease in the phosphorylated MET level was correlated with loss of total MET protein expression, and loss of other transmembrane proteins such as EGFR also occurred (Fig. 2c).

### EMT-like changes are induced in SNU-16R cell lines

**Fig. 3:**
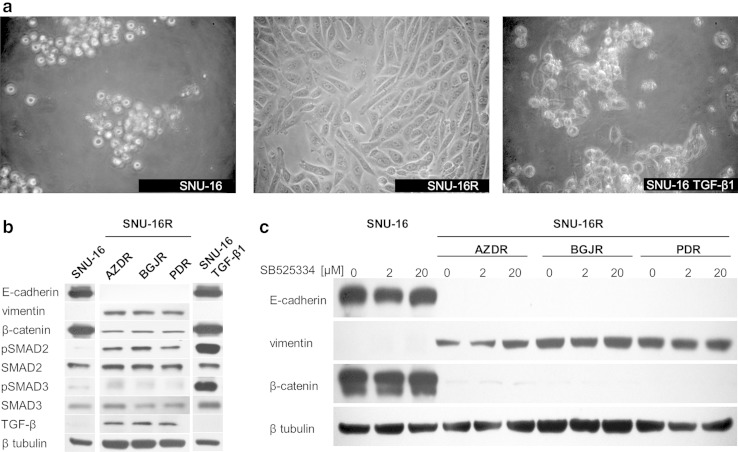
Induction of epithelial-to-mesenchymal transition (EMT) in resistant SNU-16 (*SNU-16R*) cell lines. **a** The morphology of parental SNU-16 cells, SNU-16R cells, and SNU-16 cells treated with transforming growth factor (*TGF*) β_1_ observed under a light microscope (magnification ×200). **b** Immunoblot analysis of the expression of EMT markers and TGF-β_1_ signaling proteins in parental SNU-16 cells and SNU-16R cells following incubation in inhibitor-free medium for 24 h and in parental SNU-16 cells treated with TGF-β_1_ for 3 weeks. **c** Immunoblot analysis of the expression of EMT marker proteins in parental SNU-16 and SNU-16R cell lines following incubation with the TGF-β receptor I inhibitor SB525334 for 24 h. *pSMAD2* phosphorylated SMAD2, *pSMAD3* phosphorylated SMAD3

We observed a remarkable morphologic difference between parental SNU-16 and SNU-16R cell lines. As shown in Fig. 3a, SNU-16R cells acquired a fibroblast-like, spindle-shaped morphology, suggesting that resistant cells might have undergone EMT-like changes. Recently, growing evidence has suggested that EMT is implicated in resistance to kinase-inhibitor-based therapy [24–27]. Since EMT is characterized by downregulation of epithelial phenotype markers such as E-cadherin and upregulation of mesenchymal phenotype markers such as vimentin [28], we tested the level of these markers in AZDR, BGJR, and PDR cells. This analysis revealed that E-cadherin expression was significantly reduced and vimentin expression was upregulated in all SNU-16R cell lines when compared with the parental SNU-16 cells (Fig. 3b). Additionally, we observed a substantial decrease in total β-catenin level in resistant cells, which was probably the result of acquisition of a mesenchymal phenotype associated with loss of E-cadherin/β-catenin–based adherens junctions (Fig. 3b). However, we cannot exclude intranuclear accumulation of β-catenin, a well-known EMT marker, in AZDR, BGJR, and PDR cells [29, 30]. Taken together, our findings indicate that EMT characteristics accompanied the acquisition of resistance to AZD4547, BGJ398, and PD173074.

### SNU-16R cell lines have enhanced expression of TGF-β

It has previously been shown that TGF-β signaling plays an important role in EMT, and treatment of cells with TGF-β_1_ can trigger EMT changes [24]. To verify the involvement of TGF-β signaling in EMT changes in SNU-16R cell lines, we analyzed the expression of TGF-β and activation of its downstream molecules SMAD2 and SMAD3. As shown in Fig. 3b, TGF-β expression was upregulated in all resistant SNU-16R cells, and this was accompanied by a slight increase in the phosphorylation of SMAD2.

### SNU-16 cells treated with TGF-β_1_ do not mimic EMT changes displayed in resistant cell lines

To evoke EMT in SNU-16 cells and mimic conditions which occur in in vivo tumor microenvironment [31] as well as to promote a sustained EMT and an invasive phenotype [32], cells were treated with TGF-β_1_ for 3 weeks. As shown in Fig. 3b, TGF-β_1_ treatment induced an increase in the phosphorylation level of SMAD2 and SMAD3 (Fig. 3b). However, despite observed activation of the TGF-β signaling pathway, SNU-16 cells did not acquire characteristics associated with EMT, following 3 weeks of incubation with TGF-β_1_ (Fig. 3a, b).

### EMT characteristics of SNU-16R cell lines are not abrogated by inhibition of TGF-β receptor I

**Fig. 4:**
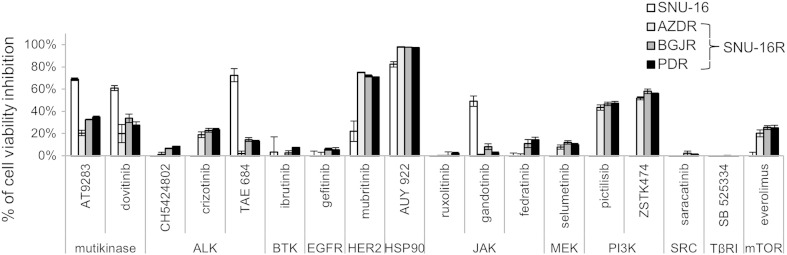
Sensitivity of parental SNU-16 and resistant SNU-16 (*SNU-16R*) cell lines to different small-molecule inhibitors. Viability of parental SNU-16 and SNU-16R cell lines following 72 h of incubation with the indicated inhibitors at 1 μM concentration. Each data point represents the mean of at least two independent experiments, and *error bars* indicate the standard deviation. *ALK* anaplastic lymphoma kinase, *AZDR* SNU-16 cell line resistant to AZD4547, *BGJR* SNU-16 cell line resistant to BGJ398, *BTK* Bruton agammaglobulinemia tyrosine kinase, *EGFR* epidermal growth factor receptor, *HER2* human epidermal growth factor receptor 2, *HSP90* heat shock protein 90, *JAK* Janus kinase, *mTOR* mammalian target of rapamycin, *PDR* SNU-16 cell line resistant to PD173074, *PI3K* phosphatidylinositol 3-kinase, *TβRI* transforming growth factor β receptor I

Owing to the essential role of TGF-β signaling in induction and maintaining EMT, TGF-β signaling pathway inhibitors are able to reverse EMT [33]. Given this, in order to evoke induction of EMT in SNU-16 cells, we treated SNU-16R cell lines with SB525334—an inhibitor of activin-receptor-like kinase 5, an isoform of TGF-β receptor I (TβRI). However, 24 h incubation with SB525334 did not reverse EMT since we did not observe an increase in E-cadherin level and a decrease in vimentin level (Fig. 3c). Additionally, SB525334 treatment for 72 h did not have any impact on the viability of all SNU-16R and parental SNU-16 cells (Fig. 4). Taken together, no EMT changes similar to those observed after long-term exposure to FGFR inhibitors were observed following TGF-β_1_ treatment.

### Mubritinib and AUY922 overcome FGFR-inhibitor-induced resistance

To identify potential alternative drug therapies for gastric cancer with EMT-mediated resistance to FGFR inhibitors, we tested the sensitivity of SNU-16R and parental SNU-16 cells to several established and investigational anticancer agents. Initially, to evaluate their impact on cell growth, the cells were treated with selected inhibitors at a concentration of 1 μM for 72 h (Fig. 4). Using this approach, we found that all SNU-16R cell lines remained sensitive to a similar extent as parental cells to the heat shock protein 90 (HSP90) inhibitor AUY922. Moreover, in contrast to the parental cell line, AZDR, BGJR, and PDR cells were shown to be more sensitive to pan-phosphatidylinositol 3-kinase inhibitors (pictilisib and ZSTK474) and a potent inhibitor of HER2 (mubritinib). However, ZSTK474 inhibited viability of parental and resistant cell lines with submicromolar IC_50_, with just slightly better efficacy against resistant cells (data not shown).

More significant from a therapeutic point of view is that mubritinib suppressed growth of the resistant cell lines at nanomolar concentrations and had little effect on the viability of the parental cell line (Fig. 4b). The calculated IC_50_ values for resistant cell lines were as follows: 146 nM for AZDR cells, 298 nM for BGJR cells, and 171 nM for PDR cells. In the case of wild-type SNU-16 cells, the IC_50_ value was not calculated since sigmoid dose–response curves could not be fitted properly to the data obtained.

**Fig. 5:**
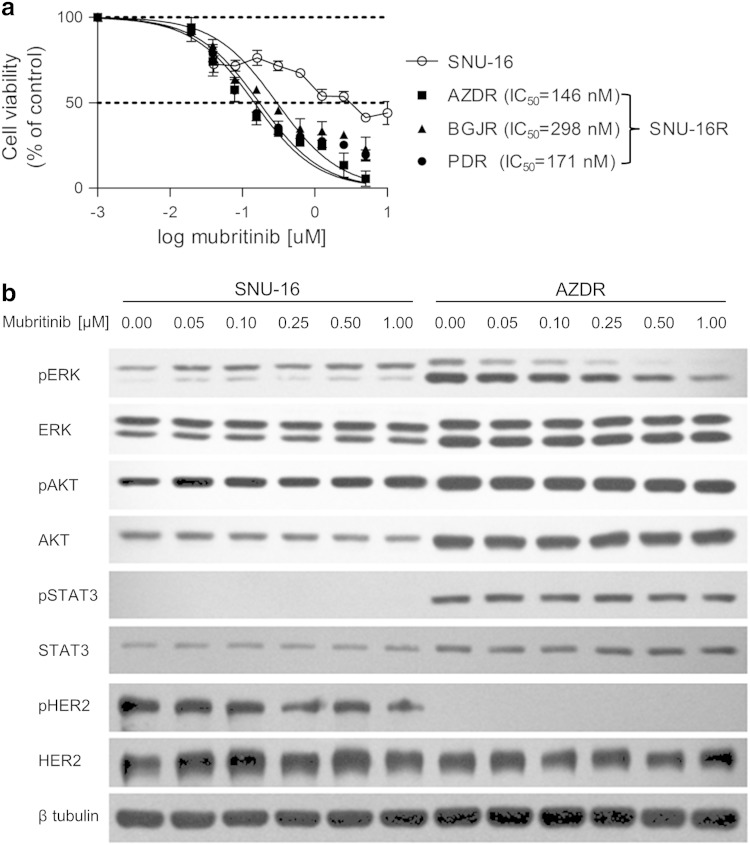
Resistant SNU-16 (*SNU-16R*) cell lines are sensitive to the human epidermal growth factor receptor 2 (*HER2*) inhibitor mubritinib. **a** Cell viability assay of a parental cell line and resistant cell lines following 72 h of incubation with increasing concentrations of mubritinib. Each data point represents the mean of two independent triplicate measurements, and *error bars* indicate the standard deviation. **b** Immunoblot analysis of protein phosphorylation status in parental SNU-16 cells and SNU-16 cells resistant to AZD4547 (*ADZR*) treated for 2 h with the indicated concentrations of mubritinib. *BGJR* SNU-16 cell line resistant to BGJ398, *ERK* extracellular-signal-regulated kinase, *PDR* SNU-16 cell line resistant to PD173074, *pERK* phosphorylated extracellular-signal-regulated kinase, *pHER2* phosphorylated HER2, *pSTAT3* phosphorylated signal transducer and activator of transcription 3, *STAT3* signal transducer and activator of transcription 3

As all three resistant cell lines displayed the same changes, we examined the effects of mubritinib treatment on the phosphorylation status of downstream proteins in AZDR cells. As shown in Fig. 5b, mubritinib reduced the level of pERK in the AZDR cell line but not in wild-type SNU-16 cells. These data suggest that mubritinib-mediated inhibition of SNU-16R cell proliferation might be the effect of suppression of ERK phosphorylation. Additionally, the activation of HER2 was markedly reduced in AZDR cells in comparison with wild-type SNU-16 cells (Figs. 2b, 5b). Thus, it is possible that the downregulation of pERK and substantial sensitivity of AZDR cells to mubritinib treatment are not the effect of HER2 inhibition. Furthermore, applying the HER2 inhibitor to SNU-16R cell lines for 48 h and 72 h did not reverse EMT since we did not observe an increase in E-cadherin level or a decrease in vimentin level (Fig. S1).

## Discussion

To predict the mechanisms of resistance to selective FGFR inhibitors, we established three resistant cell lines—AZDR, BGJR, and PDR—by long-term exposure of the human gastric cancer cell line SNU-16 to AZD4547, BGJ398, and PD173074, respectively. Compared with the parental SNU-16 cells, the resistant sublines generated were less sensitive to the FGFR inhibitors tested, with approximately two orders of magnitude higher concentration being needed to inhibit cell viability by 50 %. Moreover, they displayed cross-resistance to other FGFR inhibitors. The morphological changes in resistant cells suggested EMT, which was further confirmed by a decrease of the level of the epithelial marker E-cadherin and an increase of the level of vimentin, a mesenchymal marker. It is important to note that AZD4547, BGJ398, or PD173074, three different FGFR inhibitors of diverse selectivity and physicochemical properties, induced the same EMT changes in SNU-16 cells. This suggests that the resistance described here is specific to FGFR inhibition and does not depend on the FGFR inhibitor itself. It is highly probable that the occurrence of the resistance associated with EMT depends on the cellular context since we did not observe EMT in UM-UC-14 bladder cell lines with *FGFR3* mutation resistant to AZD4547, BGJ398, and PD173074, respectively (our unpublished data).

EMT refers to a complex molecular and cellular reorganization that in cancer is associated with metastasis, poor prognosis, and drug resistance. The changes from epithelial to mesenchymal status have been implicated in resistance to conventional and targeted anticancer drugs. It was confirmed that EMT induction confers resistance to EGFR inhibitors [26, 27], anaplastic lymphoma kinase inhibitors [34], and the multikinase inhibitor sorafenib [35]. Our study for the first time demonstrated that EMT is also a mechanism involved in acquired resistance to selective FGFR inhibitors in gastric cancer cells. At the time the manuscript was being written, Wang et al. [36] had discovered that acquired resistance to BGJ398 is characterized by EMT and a switch in dependency from FGFR to HER2/HER3 in the FGFR3-dependent bladder cancer cell line RT112. There are also some reports in the literature concerning the correlation between epithelial/mesenchymal status and sensitivity to FGFR inhibitors; however, none of them refer to acquired resistance. The multitarget FGFR inhibitor dovitinib was shown to be more effective in bladder cancer cell lines with an epithelial phenotype [37]. Furthermore, PD173074 treatment induced mesenchymal-to-epithelial transition in head and neck squamous carcinoma cells [38].

In our SNU-16-based resistance models the activation of EMT was associated with loss of FGFR2 expression. FGFR2 has previously been shown to be implicated in regulation of EMT. It was demonstrated that FGFR2IIIb, an isoform of FGFR2, occurs mainly in epithelial cells, in contrast to the FGFR2IIIc isoform, which is expressed in mesenchymal cells [39]. The switch from FGFR2IIIb to FGFR2IIIc was confirmed to promote an EMT phenotype [40]. Finally, loss of FGFR2IIIb was accompanied by loss of E-cadherin and acquisition of a mesenchymal phenotype in bladder carcinomas [41]. Our studies revealed that loss of FGFR2 expression is also implicated in resistance-associated EMT induction in SNU-16 gastric cancer cells, in which the FGFR2IIIb isoform dominates [42]. In addition to loss of FGFR2 expression, we observed reduced activation/expression of other transmembrane receptors, such as MET, HER2, HER3, and EGFR. In our FGFR-inhibitor-induced resistance models, the downregulation of these receptor kinases was accompanied by activation of SRC and downstream proteins such as ERK, AKT, and STAT3. Rho et al. [24] also observed reduced expression of EGFR, HER3, and MET and significantly enhanced activation of AKT in a gefitinib-resistant non-small-cell lung cancer cell line (A549) with EMT characteristics. However, in contrast to our results, phosphorylation of ERK was downregulated in that model [24]. The loss of expression of receptors such as FGFR2, MET, and EGFR observed here in SNU-16R cell lines suggests that another pathway is responsible for activation of downstream proteins.

It was established that TGF-β signaling is a key player in EMT-related chemotherapy and targeted therapy resistance in a number of malignancies [25, 43, 44]. TGF-β activates its signaling via binding with high-affinity type II TGF-β receptor (TβRII), which dimerizes with TβRI, which facilitates TβRI phosphorylation. The activation of TβRI leads to the propagation of signaling by the canonical SMAD-dependent pathway and the noncanonical SMAD-independent pathway. The mesenchymal features and increase in phosphorylation level of proteins such as AKT, ERK, and SRC suggested activation of noncanonical TGF-β signaling in SNU-16R cell lines [45, 46]. However, we failed to induce EMT changes in parental SNU-16 cells by TGF-β_1_ treatment. The only evidence of the induction of the TGF-β signaling pathway in the resistant cells was upregulated TGF-β ligand expression and slight activation of SMAD2. These differences between TGF-β_1_-treated parental SNU-16 cells and SNU-16R cells suggest that, in addition to TGF-β-activated signaling, other unidentified pathways might be responsible for induction of an EMT-like phenotype of SNU-16R cells. A similar conclusion was drawn by Suda et al. [47], who also failed to prove the involvement of TGF-β in acquired resistance to erlotinib. Moreover, applying SB525334—an inhibitor of TβRI—to SNU-16R cell lines neither reversed EMT nor reduced cell viability. Our results suggest that the application of a single inhibitor of one of the isoforms of TβRI is not sufficient to reverse EMT-like changes in SNU-16R cell lines.

Since we did not observe any impact of the TβRI inhibitor on the viability of the SNU-16R cell lines, we decided to test other known anticancer agents in order to determine an alternative therapeutic strategy for overcoming EMT-mediated resistance to selective FGFR inhibitors. We found that SNU-16R cells remained sensitive to the HSP90 inhibitor AUY922. In support of our results, HSP90 inhibitors effectively suppress growth of crizotinib-resistant H2228 lung cancer cells with EMT changes [34]. Kim et al. [34] suggested that the overcoming of resistance by HSP90 inhibitors resulted from reversal of EMT due to degradation of TβRII and further the restoration of E-cadherin expression. Finally, we found that an inhibitor of HER2 can overcome FGFR inhibitor resistance mediated by the EMT since all the SNU-16R cell lines, in contrast to parental cells, were highly sensitive to mubritinib. Moreover, the observed marked sensitivity of resistant cells to mubritinib suggests that use of this inhibitor might be an alternative therapeutic strategy for the management of gastric cancer resistant to FGFR inhibitors owing to EMT. The exact mechanism of mubritinib-mediated growth inhibition of the SNU-16R cell lines is unclear, and it might be the effect of inhibition of targets other than HER2, since we observed a decrease in activation of HER2 in SNU-16R cells, in contrast to results reported by Wang et al. [36]. Furthermore, unlike in the wild-type SNU-16 cells, mubritinib treatment of AZDR cells suppressed phosphorylation of ERK. However, further investigations are needed to clarify the mechanism of action of mubritinib in the SNU-16R cells. In conclusion, we found that EMT was induced as a result of long-term exposure of a gastric cancer cell line in vitro to selective FGFR inhibitors. Additionally, EMT induction was associated with reduction in expression of FGFR2 and other transmembrane receptors. On the basis of our study, EMT should be considered as a possible acquired resistance mechanism contributing to the decreased efficacy of FGFR-inhibitor-based therapy in cancer patients. Furthermore, our data suggest that the use of mubritinib or AUY922 treatment may be an alternative therapeutic approach for treating cancer patients with EMT-mediated resistance to FGFR inhibitors.

## Electronic supplementary material

Below is the link to the electronic supplementary material.

Supplementary material 1 (TIFF 4623 kb)

Supplementary material 2 (DOCX 15 kb)
