# Supplementary figures and images for: Epithelial–mesenchymal transition confers resistance to selective FGFR inhibitors in SNU-16 gastric cancer cells
Source: Gastric Cancer. 2014 Nov 19;19(1):53–62. doi: 10.1007/s10120-014-0444-1 (PMC4688307; doi:10.1007/s10120-014-0444-1)

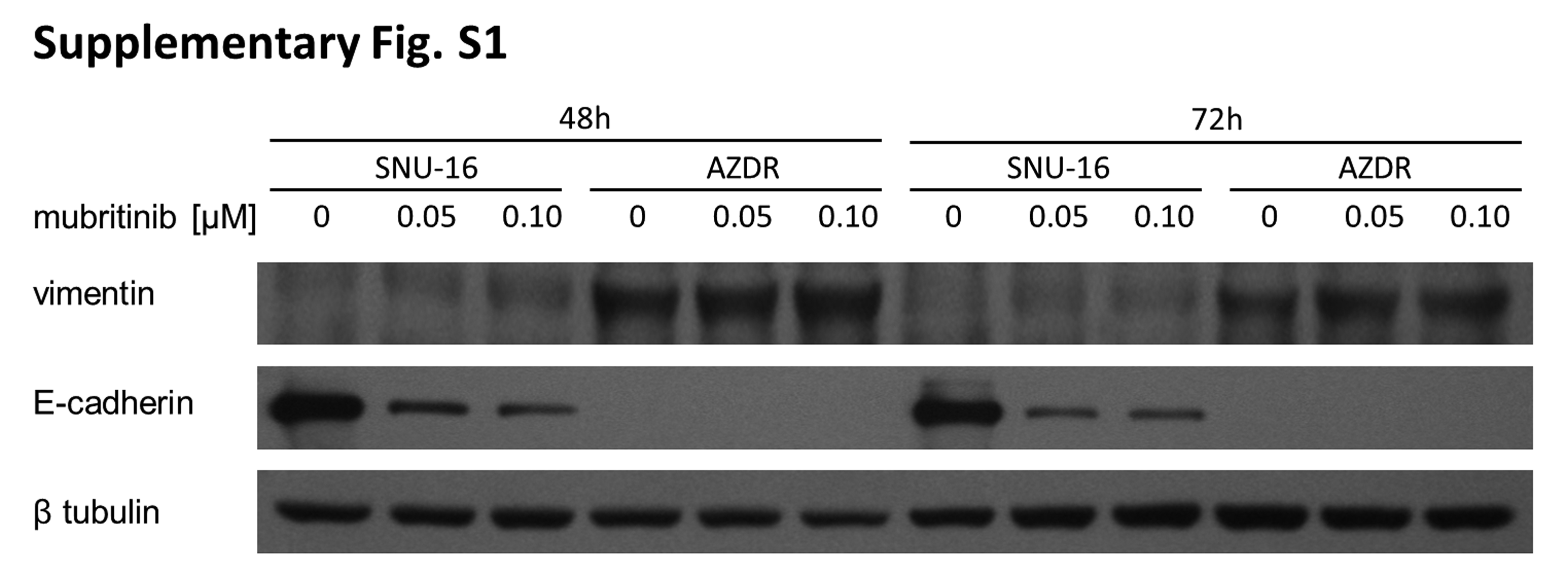

Supplement: Supplementary file 1 — Supplementary material 1 (TIFF 4623 kb) [file 10120_2014_444_MOESM1_ESM.tif]
