# Supplementary material for: Epithelial–mesenchymal transition confers resistance to selective FGFR inhibitors in SNU-16 gastric cancer cells
Source: Gastric Cancer. 2014 Nov 19;19(1):53–62. doi: 10.1007/s10120-014-0444-1 (PMC4688307; doi:10.1007/s10120-014-0444-1)
Supplement: Supplementary file 2 — Supplementary material 2 (DOCX 15 kb) [file 10120_2014_444_MOESM2_ESM.docx]

**Supplementary Materials**

Journal: **Gastric Cancer**

Article title: **Epithelial-mesenchymal transition confers resistance to selective FGFR inhibitors in gastric cancer SNU-16 cells**

Short Title:  **EMT confers resistance to FGFR-TKI**

Paulina Grygielewicz^a,b^*, Barbara Dymek^a^, Anna Bujak^a,b^, Pawel Gunerka^a^, Aleksandra Stanczak^a^, Monika Lamparska-Przybysz^a^, Maciej Wieczorek^a^, Karolina Dzwonek^a,c^ and Daria Zdzalik^a^

^a^Innovative Drugs R&D Department, Celon Pharma Inc., Mokra 41a, 05-092 Lomianki/Kielpin, Poland

^b^Postgraduate School of Molecular Medicine, Zwirki i Wigury 61, 02-091 Warsaw, Poland

^c^Department of Immunology, Center for Biostructure Research, Medical University of Warsaw, Banacha 1a, F Building, 02-097 Warsaw, Poland

***To whom correspondence should be addressed:**

Paulina Grygielewicz, Innovative Drugs R&D Department, Celon Pharma Inc., Mokra 41a, 05-092 Lomianki/Kielpin, Poland

Tel.: +48665646589 Fax: +48 227517477 Email: [paulina.grygielewicz@celonpharma.com](mailto:paulina.grygielewicz@celonpharma.com)

**Supplementary figure legend**

**Supplementary Fig. S1** EMT characteristics of AZDR cells is not reversed by mubritinib treatment
Immunoblot analysis of the expression of EMT marker proteins in parental SNU-16 and resistant AZDR cell lines following incubation with HER2 inhibitor- mubritinib for 48 and 72 hours.
